# A large-scale comparison of clinical outcomes to IBD therapies in White and South Asian ethnicities

**DOI:** 10.1016/j.eclinm.2025.103644

**Published:** 2025-11-18

**Authors:** Sharmili Balarajah, Laura Martinez-Gili, James L. Alexander, Benjamin H. Mullish, Robert W. Perry, Jia V. Li, Julian R. Marchesi, Miles Parkes, Timothy R. Orchard, Lucy C. Hicks, Horace R.T. Williams, UK IBD BioResource Investigators

**Affiliations:** a Department of Metabolism, Digestion and Reproduction, Imperial College London, UK; b Departments of Gastroenterology and Hepatology, Imperial College Healthcare NHS Trust, UK; c IBD Unit, St Mark's National Bowel Hospital, London, UK; d Department of Medicine, University of Cambridge, UK; e Department of Gastroenterology, Cambridge University Hospitals NHS Trust, UK

**Keywords:** IBD, UC, CD, Treatment, Outcomes, Ethnicity, Adverse events

## Abstract

**Background:**

While ethnic differences in IBD phenotype are recognised, the comparative efficacy and safety of common IBD therapies across ethnically diverse populations remain uncertain. This multicentre cohort study aimed to compare the efficacy and safety of these therapies between White (WH) and South Asian (SA) IBD cohorts.

**Methods:**

Demographic, phenotypic and outcome data from the UK IBD BioResource were utilised (BioResource inception date: 1st January 2016; data lock for analysis: 12th May 2023). The primary outcome was treatment response (defined as treatment persistence free of discontinuation or failure) to 5-aminosalicylates (5-ASAs), thiopurines and anti-TNFs. The secondary outcome was the occurrence of treatment-related adverse events (AEs). Ethnic differences in treatment response were evaluated using propensity score weighting and Cox proportional hazards regression. AE occurrence was assessed through logistic regression analysis.

**Findings:**

In total, 26,530 patients were included [51.2% females; median age at diagnosis 30 years (IQR 21–43); 96.1% WH, 3.9% SA]. SA were diagnosed at a younger age, began treatment younger than WH, and demonstrated baseline phenotypic differences including more perianal disease in CD. However, no significant differences in treatment response between WH and SA were identified in either CD (reference group WH; thiopurines, HR 0.82 (95% CI 0.53–1.27), p = 0.37; anti-TNFs, HR 1.07 (95% CI 0.34–3.37), p = 0.91] or UC [thiopurines, HR 0.98 (95% CI 0.95–1.02), p = 0.36; anti-TNFs, HR 0.98 (95% CI 0.92–1.04), p = 0.54]. SA were at significantly increased risk of pancreatitis [HR 2.34 (95% CI 1.37–3.75), p = 0.001] and leucopenia [HR 1.76 (95% CI 1.02–2.84), p = 0.03] with thiopurines, and renal dysfunction with anti-TNFs [HR 4.75 (95% CI 1.55–12.07), p = 0.002].

**Interpretation:**

Treatment efficacy in similar WH and SA IBD patients recruited to the UK IBD BioResource is unaffected by ethnicity but patients from SA ethnic backgrounds are at increased risk of developing pancreatitis and leucopenia with thiopurines, and renal dysfunction with anti-TNFs. These findings highlight the importance of comprehensive risk assessment and counselling by clinicians, and emphasise the importance of improving ethnic representation in IBD research.

**Funding:**

Bowel Research UK; 10.13039/501100013342NIHR Imperial Biomedical Research Centre (BRC); 10.13039/501100018956NIHR Cambridge BRC.

**Research in context**

**Evidence before this study**

To date, clinical trials in IBD therapies have been conducted in White-predominant populations. However, there are ethnicity-related phenotypic differences in IBD, and ethnic variations in medication-induced adverse events. There have been no studies investigating ethnic differences in response to 5-aminosalicylates (5-ASAs), thiopurines, and very few to anti-tumour necrosis factor agents (anti-TNFs), with inconsistent results.

**Added value of this study**

To our knowledge, this is the first large-scale cohort study to investigate ethnicity-related outcomes of 5-ASA, thiopurine and anti-TNF therapy. These treatments demonstrated comparable efficacy in White (WH) and South Asian (SA) patients; however, SA patients exhibited an increased risk of adverse events, including pancreatitis and leucopenia with thiopurines, and renal dysfunction with anti-TNFs.

**Implications of all the available evidence**

Reassuringly, there were no significant differences in treatment efficacy between similar WH and SA cohorts. However, there was a greater risk of adverse events in SA patients. Clinicians must be aware of this when prescribing medications in this context. Additionally, this work highlights the vital need to improve ethnic minority representation in clinical trials, to facilitate deeper insights into treatment effects across diverse patient groups.

## Introduction

The treatment of IBD has rapidly evolved over the past 40 years, and the introduction of biologics has further revolutionised IBD management. However, clinical trials for most IBD therapies have been conducted in White-predominant populations.^1^, ^2^, ^3^ Within the UK, SA make up the largest proportion of the minority population.^4^ Research has demonstrated ethnicity-related phenotypic differences^5^^,^^6^ and ethnic variations in medication-induced adverse events (AEs), such as the increased risk of thiopurine-induced leucopenia.^7^ Given the limited ethnic representation in clinical trials and the known impact of ethnicity on treatment tolerance, it is crucial to consider the potential for differential response to therapies in ethnically-diverse cohorts.

There have been no studies investigating response to 5-aminosalicylates (5-ASAs) and thiopurines in SA. Ethnic differences in anti-tumour necrosis factor agent (anti-TNF) response have been assessed in a small retrospective cohort from a London tertiary centre,^8^ and pooled data analyses of infliximab (IFX)^9^ and golimumab (GOL)^10^ trials. The findings from these studies, however, are limited by small non-White sample sizes^8^^,^^10^ and restricted statistical analyses,^8^ with inconsistent results.

The UK IBD BioResource (IBD-BR) is an open-access platform established by the National Institute of Health Research (NIHR) in 2016.^11^ The database component collects key demographic and clinical details, and has been used to demonstrate differential efficacy of thiopurines between CD and UC, as well as the superiority of first-line anti-integrins over anti-TNFs in UC.^12^^,^^13^

In this large-scale cohort study, we sought to investigate ethnicity-related outcomes to 5-aminosalicylate (5-ASAs), thiopurine, and anti-TNF therapy using the IBD-BR. We hypothesise differences in the efficacy and risk of developing adverse events to therapies between White (WH) and South Asian (SA) cohorts.

## Methods

### Database

Phenotypic and outcome data were extracted from the NIHR IBD-BR (REC reference 15/EE/0286, IRAS 173561). All participants provided written consent. Access to the NIHR IBD-BR was approved by the NIHR IBD-BR Steering Committee (approval reference number: DAA060). Structured demographic, phenotypic and treatment and outcome data were ascertained at the time of enrolment through a combination of clinical records review, patient interview, and patient questionnaire. Pre-enrolment data were collected retrospectively, while subsequent data were captured prospectively during routine clinical appointments and annual interviews. Data validity is upheld through regular validation procedures, manual data standardisation, and random case audits.^11^

### Eligibility criteria

A total of 35,802 patients had been enrolled at the time of data extraction (12th May 2023) (no formal power calculation was performed for this study, but all patients within the NIHR IBD-BR were considered). Participants with a confirmed diagnosis of either CD or UC, and of WH and SA ethnicity were included. SA ethnicity was defined as Bangladeshi, Indian, and Pakistani ethnic subgroups as per the Government Statistical Service and Statistician Group harmonised standard.^14^ Patients with IBD unclassified, of any other ethnic background, or diagnosed before 1960 (earliest year of IBD diagnosis in the non-WH cohort) were excluded.

Insufficient patient numbers prevented the assessment of response to first-line anti-integrins (CD, WH n = 335, SA n = 11; UC, WH n = 716, SA n = 36) and Ustekinumab (CD, WH n = 86, SA n = 1; UC, WH n = 17, SA n = 0).

### Statistics

All statistical analyses were conducted in R (version 4.3.2).^15^ Non-parametric variables were reported as median (interquartile range), and continuous parametric variables as mean (standard deviation). Demographic and phenotypic characteristics were summarised using descriptive statistics: Chi-square testing for categorical variables, and Mann Whitney U for continuous variables.

The primary outcome assessed was treatment response to 5-ASAs (UC only), thiopurines (azathioprine or mercaptopurine) and anti-TNFs [IFX, adalimumab (ADA), GOL (UC only)]. The secondary outcome assessed was the risk of AEs to these therapies.

#### Treatment response

Patients were excluded from treatment response analysis if start dates for the treatment being assessed were missing; therapy was commenced within 12 months of surgery (CD, to exclude prophylactic therapy), or if a colectomy had occurred prior to commencing therapy (UC).

Follow-up began at the time of commencing the drug being assessed, and continued until treatment failure. If being treated with a 5-ASA or thiopurine, treatment failure was defined as surgery (defunctioning or resection in CD, and colectomy in UC), and escalation to biologics. If being treated with an anti-TNF, treatment failure was defined as surgery (see above) and switching to another biologic. In addition to treatment start and stop dates, the IBD-BR also documents treatment outcomes (categorised into eight variables; see Supplementary Text). These data were utilised to corroborate the ‘treatment persistence’ definition of treatment effectiveness. Failure-free samples were censored at the time of treatment discontinuation or data extraction.

Propensity score weighting targeting the average treatment effect in the overlap population (ATO) was performed to account for imbalance between WH and SA groups using the *WeightIt* R package.^16^ The ATO was chosen due to its robustness and low bias, as it prioritised comparisons among WHs and SAs who were most similar to each other.^17^ Weights were generated with the overlap weighting method,^18^ using propensity scores from a logistic regression including variables selected *a priori* due to their clinical relevance: age at diagnosis, age at commencing therapy, sex, smoking status, disease extent, diagnostic era for 5-ASAs and thiopurines (see Supplementary Text), disease behaviour and perianal disease (CD), concurrent corticosteroid use and adverse events (disease flares excluded) with the therapy being assessed. Covariable balance after weighting was confirmed as being below 0.1 mean difference, and the effective sample size constituted 99% of SA and 68% of WH on average. Cox regression analysis was conducted with the coxph_weighit function from the *WeightIt* R package^16^ to assess for differences in treatment response between ethnic groups. Standard errors and confidence intervals were calculated utilising the bootstrap method,^19^ and adjusted survival curves plotted using the *adjustedCurves* R package.^20^ Additional sensitivity analyses for all treatments were performed, excluding patients with adverse events to the treatment being assessed. For these and any subgroup analyses, groups were reweighted prior to the application of Cox regression.

Additional sensitivity analyses, excluding patients who experienced adverse events, were conducted for all treatments, and involved reweighting groups prior to applying Cox regression analyses. Additionally, for any subgroup analyses, groups were also reweighted before Cox regression analysis was applied.

Cox regression analysis without weighting was also conducted to identify variables other than ethnicity that may affect treatment response. This analysis adjusted for age at commencing therapy, sex, smoking status, ethnicity, presence of co-morbidities, history of extraintestinal manifestations (EIMs), disease extent, diagnostic era for 5-ASAs and thiopurines, disease behaviour and perianal disease (CD), time from diagnosis to commencing therapy, adverse event with therapy, concurrent corticosteroid use and concomitant immunomodulator use (anti-TNFs). Adverse events did not include disease flares. These variables were selected *a priori* and assessed for co-linearity.

#### Adverse events

Occurrence of adverse events for each treatment was recorded as “yes” or “no” for each pre-specified event in the IBD-BR data collection (see Supplementary Tables S9–S11), and were assessed in all patients on first exposure to any of the therapies being assessed (as above). With no dates available for adverse events in the IBD-BR, logistic regression analysis was conducted to identify variables associated with the occurrence of adverse events: age at commencing therapy, ethnicity, sex, smoking status at the time of diagnosis, IBD subtype and the presence of co-morbidities and EIMs. Disease flares were excluded.

### Role of funding source

The funders played no role in the writing of the manuscript nor the decision to submit for publication. Authors received no payment for writing this manuscript.

## Results

**Fig. 1:**
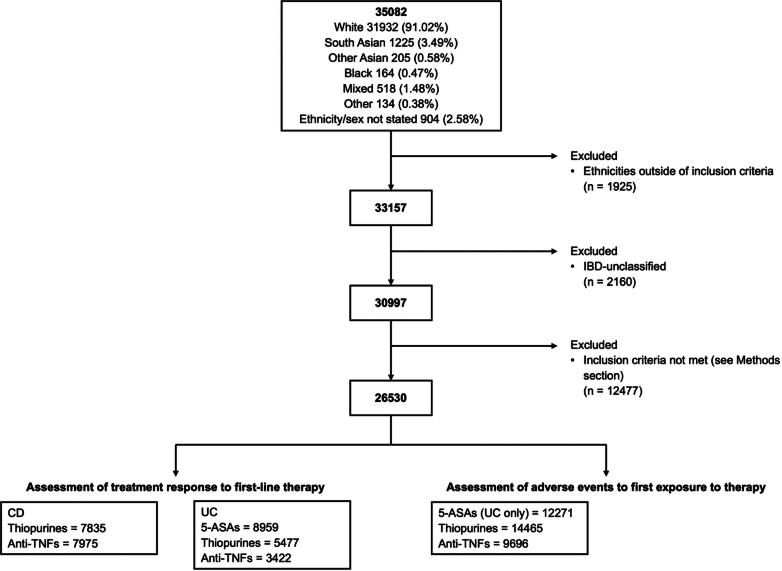
Study flow chart.

A total of 26,530 patients were included [13,574 (51.2%) females; median age at diagnosis 30 years (IQR 21–43)]; 96.1% WH and 3.9% SA. 12,665 CD (97.2% WH, 2.8% SA) and 13,865 UC (95.2% WH, 4.8% SA) were assessed (Fig. 1).

### Treatment response

#### 5-ASAs (UC only)

A total of 8959 patients (95.3% WH, 4.7% SA) received 5-ASA therapy. Baseline characteristics of these patients can be seen in Supplementary Table S1. SA were younger at 5-ASA initiation compared to WH patients, and the time from diagnosis to 5-ASA initiation was similar between groups.

**Fig. 2:**
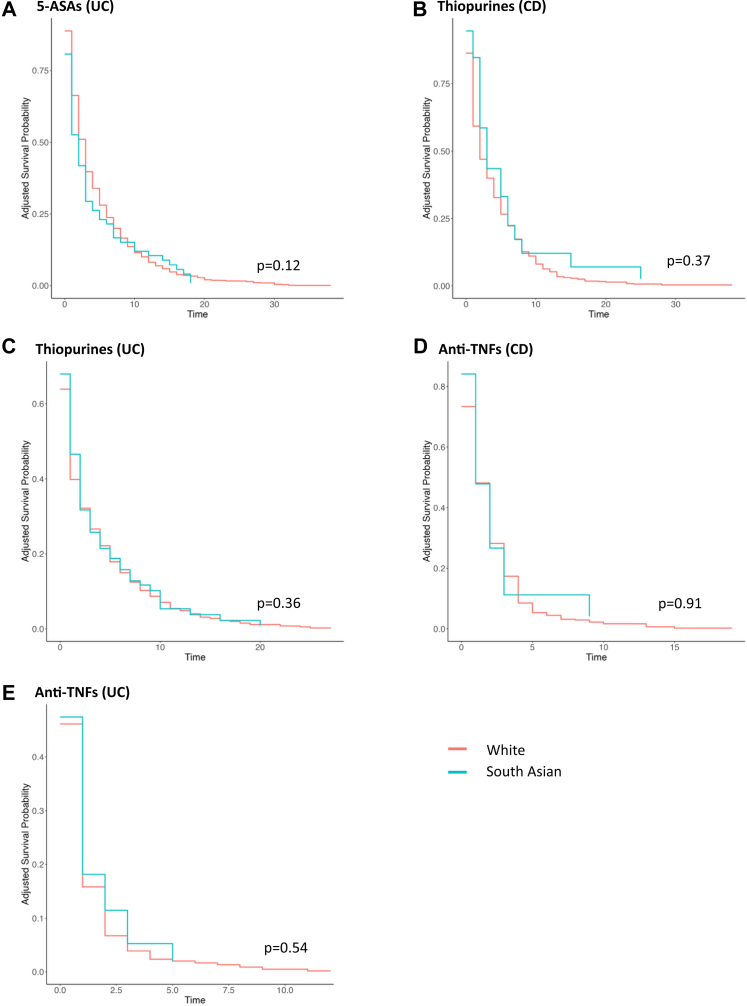
Adjusted survival curves for assess therapies in White and South Asian IBD. A) 5-ASAs (in UC); B) Thiopurines (in CD); C) Thiopurines (in UC); D) Anti-TNFs (in CD); E) Anti-TNFs (in UC). 5-ASA, 5-aminosalicylic acid; CD, Crohn's disease; IBD, inflammatory bowel disease; TNF, tumour necrosis factor; UC, ulcerative colitis.

Response to 5-ASAs was not influenced by ethnicity [HR 1.20 (95% CI 0.96–1.50), p = 0.12; Fig. 2A]. A sensitivity analysis, which excluded individuals experiencing an adverse event to 5-ASAs, also showed no link between ethnicity and treatment response [HR 1.13 (95% CI 0.90–1.41), p = 0.28]. A further analysis was conducted in just those with extensive disease given the higher risk of colectomy in this cohort,^21^ and no association between ethnicity and treatment response [HR 1.25 (95% CI 0.87–1.79), p = 0.23] was identified.

Older age at commencing 5-ASAs and developing an adverse event with 5-ASAs were also associated with an increased risk of treatment failure (Supplementary Table S7).

#### Thiopurines (monotherapy)

A total of 13,282 patients had received thiopurine monotherapy [CD, total 7835, WH 97.1%, 2.9% SA; UC, total 5447, WH 94.5%, 5.5% SA; see Supplementary Tables S2 and S3 for baseline characteristics). SA were younger to commence thiopurine monotherapy than WH.

##### Response in CD

SA ethnicity was not associated with an increased risk of treatment failure [HR 0.82 (95% CI 0.53–1.27), p = 0.37; Fig. 2B]. Analysis of a subset of patients without a thiopurine-related adverse event revealed no association between ethnicity and treatment response [HR 0.82 95% CI (0.47–1.47), p = 0.52].

Older age at commencing therapy, increased duration from diagnosis to treatment initiation and developing an adverse event to thiopurines were all associated with an increased risk of treatment failure (Supplementary Table S6).

##### Response in UC

SA ethnicity was not associated with a difference in treatment response compared to WH [HR 0.98 (95% CI 0.95–1.02), p = 0.36; Fig. 2C]. On further sensitivity analyses conducted in only those who did not experience an adverse event, there remained no ethnic difference in treatment response [HR 0.99 (95% CI 0.94–1.04), p = 0.73]. When treatment response was assessed exclusively in individuals with extensive disease, ethnicity was not linked to treatment response [HR 0.99 (95% CI 0.94–1.04), p = 0.59].

Similar to CD, older age at commencing therapy, increased duration from diagnosis to treatment initiation and developing an adverse event to thiopurines were associated with an increased risk of treatment failure in UC (Supplementary Table S7).

### Anti-TNFs

A total of 11,397 patients were treated with anti-TNFs as the first-line biologic (CD, total 7975, WH 97.0%, SA 3.0%; UC, total 3422, WH 94.0%, SA 6.0%). In CD, 64.2% of patients received first-line IFX, while 35.8% received ADA. In UC, 71.5% received first-line IFX, 24.6% ADA, and 3.9% GOL. Baseline characteristics are detailed in Supplementary Tables S4 and S5

#### Response in CD

SA ethnicity was not associated with anti-TNF failure [HR 1.07 (95% CI 0.34–3.67), p = 0.91; Fig. 2D]. On sensitivity analyses, assessing only those who did not develop an adverse event to anti-TNF therapy, there remained no association between ethnicity and treatment response [HR 1.11 (95% CI 0.26–4.84), p = 0.89]. Additionally, on assessing just those with perianal involvement, no association between ethnicity and treatment response was identified [HR 1.10 (95% CI 0.67–1.80), p = 0.70].

Concomitant immunomodulator use and a history of EIMs was associated with a reduced risk of treatment failure whilst developing an adverse event to anti-TNF therapy was associated with an increased risk (Supplementary Table S6).

#### Response in UC

Ethnicity was not associated with a difference in anti-TNF response [HR 0.98 (95% CI 0.92–1.04), p = 0.54; Fig. 2E) nor on analysis restricted to patients who did not experience an adverse event to anti-TNFs [HR 0.95 (95% CI 0.86–1.05), p = 0.35]. On assessment limited to those with extensive disease, there remained no association between ethnicity and treatment response [HR 1.02 (95% CI 0.92–1.13), p = 0.66]. On independently assessing IFX and ADA in SA (compared to WH), SA ethnicity was not associated with a difference in treatment response [IFX, HR 0.98 (95% CI 0.91–1.05), p = 0.55; ADA, HR 0.97 (95% CI 0.10–9.34), p = 0.98].

Concomitant immunomodulator use and reduced time from diagnosis to treatment initiation were associated with a reduced risk of treatment failure whilst an older age at commencing anti-TNF therapy was associated with an increased risk (Supplementary Table S7).

### Adverse events (AEs)

#### 5-ASAs

A total of 12,271 patients (WH 95.2%, SA 4.8%) had been treated with 5-ASAs (baseline characteristics summarised in Supplementary Table S8). The prevalence of adverse events is summarised in Supplementary Table S9. SA ethnicity (compared to WH) was not associated with an increased risk of experiencing an adverse event with 5-ASAs [SA, OR 1.06 (95% CI 0.76–1.44), p = 0.73] but older age at commencing a 5-ASA [OR 1.01 (95% CI 1.00–1.01), p ≤ 0.001], female sex [OR 1.17 (95% CI 1.02–1.35), p = 0.03] and a history of EIMs [OR 1.23 (95% CI 1.00–1.51), p = 0.046; Table 1] were.

**Table 1:** Logistic regression analysis of risk of adverse events in White (WH) and South Asian (SA) IBD with 5-ASAs, thiopurines and anti-TNFs.

	5-ASA (UC only)	Thiopurines	Anti-TNFs
	WH n = 11,688 SA n = 583	WH n = 13,905 SA n = 560	WH n = 9324 SA n = 372
	OR (95% CI), p	OR (95% CI), p	OR (95% CI), p
**Age at commencing therapy**	1.01 (1.00–1.01), <0.001^∗∗∗^	1.02 (1.01–1.02), <0.001^∗∗∗^	1.00 (1.00–1.01), 0.13
**Ethnicity**			
White (reference group)	_	_	_
South Asian/Black	1.06 (0.76–1.44), 0.73	1.33 (1.08–1.62), 0.006^∗∗^	1.40 (0.98–1.95), 0.05
**Sex**			
Male (reference group)	_	_	_
Female	1.17 (1.02–1.35), 0.03^∗^	1.27 (1.18–1.37), <0.001^∗∗∗^	1.94 (1.68–2.24), <0.001^∗∗∗^
**IBD subtype**			
CD (reference group)	_	_	_
UC	_	0.93 (0.85–1.01), 0.07	0.95 (0.81–1.10), 0.48
**Smoking status at diagnosis**			
Never smoker (reference group)	_	_	_
Ex-smoker	0.80 (0.69–0.93), 0.004^∗∗^	1.09 (0.99–1.19), 0.07	1.02 (0.87–1.20), 0.78
Current smoker	0.89 (0.65–1.18), 0.43	1.21 (1.07–1.36), 0.002^∗∗^	1.18 (0.96–1.44), 0.12
**Co-morbidity**	1.01 (0.86–1.18), 0.92	1.01 (0.93–1.11), 0.79	1.27 (1.08–1.48), 0.003^∗∗^
**History of EIMs**	1.23 (1.00–1.51), 0.046^∗^	1.25 (1.13–1.38), <0.001^∗∗∗^	1.58 (1.34–1.84), <0.001^∗∗∗^

^∗^p < 0.05, ^∗∗^p < 0.001, ^∗∗∗^p < 0.001.

WH, White; SA, South Asian; EIMs, extraintestinal manifestations.

Adjusted for age at commencing therapy, ethnicity, sex, smoking status, IBD subtype, co-morbidities and EIMs.

### Thiopurines

14,465 patients (WH 96.1%, SA 3.9%) were exposed to thiopurines during their disease course (see Supplementary Table S8 for baseline characteristics). Supplementary Table S10 presents a breakdown of the occurrence of adverse events. SA were at increased risk of experiencing any form of adverse event with thiopurines compared to WH [OR 1.33 (95% CI 1.08–1.62), p = 0.006]. Other factors associated with experiencing an adverse event with thiopurines were older age at commencing the drug [OR 1.02 (95% CI 1.01–1.02), p < 0.001], female sex [OR 1.27 (95% CI 1.18–1.37), p < 0.001], a history of smoking at diagnosis [OR 1.21 (95% CI 1.07–1.36), p = 0.002], and a history of EIMs [OR 1.25 (95% CI 1.13–1.38), p ≤ 0.001; Table 1). Subsequent analysis of individual adverse events demonstrated that SA ethnicity was associated with an increased risk of pancreatitis [OR 2.34 (95% CI 1.37–3.75), p = 0.001] and leucopenia [OR 1.76 (95% CI 1.02–2.84), p = 0.03].

### Anti-TNFs

A total of 9696 patients had a history of exposure to anti-TNFs (WH 96.2%, SA 3.8%; see Supplementary 8 for baseline features). The prevalence of adverse events in each group is tabulated in Supplementary Table S11. A trend towards an increased risk of experiencing an AE in SA with anti-TNF therapy was observed [OR 1.40 (0.98–1.95), p = 0.05]. Female sex [OR 1.94 (95% CI 1.68–2.24), p < 0.001], the presence of co-morbidities [OR 1.27 (95% CI 1.08–1.48), p = 0.003], and a history of EIMs [OR 1.58 (95% CI 1.34–1.84), p < 0.001] were also associated with an increased risk of experiencing an adverse event with anti-TNF therapy (Table 1). Subsequent analysis of individual adverse events revealed that SA ethnicity was associated with an increased risk of renal dysfunction [OR 4.75 (95% CI 1.55–12.07), p = 0.002].

## Discussion

Minority ethnic groups have historically been underrepresented in drug efficacy and safety trials, though ethnic differences in IBD phenotype have been demonstrated.^5^^,^^6^ In the first and largest study of its kind, the UK IBD-BR has been used to demonstrate that SA ethnicity is not associated with a difference in response to IBD therapies (5-ASAs, thiopurines and anti-TNFs), but is associated with the risk of experiencing an AE to these therapies.

To date, no studies have investigated the efficacy of 5-ASAs and thiopurines in ethnically-diverse populations, and few have done so with anti-TNFs.^8^, ^9^, ^10^ Reassuringly, we have found no ethnicity-related differences in the efficacy of thiopurines and anti-TNFs. A review of randomised placebo-controlled trials to assess for racial disparities in IFX efficacy in UC demonstrated that IFX efficacy was independent of ethnicity,^9^ corroborating our findings. However, a retrospective single-centre cohort study of UK Bangladeshi (n = 54) and White CD patients being treated with anti-TNFs suggested that Bangladeshis were less responsive to anti-TNFs.^8^ This discrepancy in findings may be explained by the lack of adjustment for smoking and sex, both of which are known to influence outcomes in CD.^22^^,^^23^ Greywoode et al. specifically demonstrated racial differences in the efficacy of GOL in UC,^10^ however, only 3.9% of our UC cohort received GOL.

We found that developing an adverse event to a given therapy increases the risk of treatment failure, and in turn, SA are at higher risk of developing an adverse event when treated with 5-ASAs, thiopurines and anti-TNFs. Studies have previously identified an elevated risk of adverse events among ethnic minority groups with non-IBD medications. Examples include clomipramine in South Asians and ACE inhibitors in African Americans.^24^ The increased risk of adverse events in the SA cohort may be attributed to the complex interplay between genetic variations and environmental factors.^24^ Among individual AEs, SA had a higher risk of pancreatitis (thiopurines), leucopenia (thiopurines) and renal dysfunction (anti-TNFs). Thiopurine-induced leucopenia in Asian populations has previously been noted.^25^ However, the role of ethnicity in thiopurine-induced pancreatitis remains unexplored, and while anti-TNF therapy has been associated with renal dysfunction,^26^ there have been no studies examining the impact of ethnicity in this context. Genetic polymorphisms such as the HLA-DQA1-HLA-DRB1 variants (shown to confer susceptibility to thiopurine-induced pancreatitis^27^), pharmacokinetic differences and differences in the microbiome may explain these findings,^28^^,^^29^ however further research is required.

Female sex was also found to be independently associated with the occurrence of adverse events in all assessed drugs, with an increased risk of treatment failure, highlighting the need to improve representation of females in clinical trials assessing drug effectiveness and dosing.

Our findings were derived from a national dataset; more than 26,000 patients were studied. Other strengths include length of follow up, use of propensity score weighting to compare treatment response between ethnic groups, extensive efforts to adjust for confounders, and broad coverage of a spectrum of patients from district general and tertiary centres. However, we acknowledge that our findings relate to the population of SA with IBD which has been recruited to the UK IBD BioResource, which may be prone to unknown biases. We also acknowledge other potential limitations. Due to lack of data, we could not assess drug and anti-drug antibody levels and for biochemical evidence of treatment response, nor were we able to account for socioeconomic variables and adherence to therapy. Nevertheless, the impact of socioeconomic factors may be partly mitigated by the equitable treatment approach within the UK National Health Service, which gives all IBD patients access to the full spectrum of therapies according to clinical need.^6^ Additionally, we were unable to assess ethnic differences in response to anti-integrins and Ustekinumab due to the small number of SA patients in these treatment cohorts, and amongst other non-White ethnic groups due to the small number of patients in these cohorts.

In conclusion, this large-scale study demonstrates that treatment response to 5-ASAs, thiopurines and anti-TNFs is not associated with ethnicity. However, we have shown that SA patients are at increased risk of pancreatitis and leucopenia with thiopurines, and renal dysfunction with anti-TNFs. This research emphasises the importance of risk assessment and comprehensive counselling by clinicians. Furthermore, our study emphasises the critical need for improved ethnic representation in clinical trials to facilitate a deeper insight into treatment effects across diverse groups.

## Contributors

Sharmili Balarajah: conceptualisation; data curation; formal analysis; investigation; methodology; project administration; visualisation; writing–original draft. Laura Martinez-Gili: data curation; formal analysis; investigation; methodology; resources; software; visualisation; writing–original draft. James L Alexander: investigation; project administration; resources; supervision; writing–review and editing. Benjamin H Mullish: investigation; project administration; resources; supervision; writing–review and editing. Robert W Perry: writing–review and editing; Jia V Li: investigation; methodology; supervision; writing–review and editing; Julian R Marchesi: investigation; methodology; project administration; resources; supervision; writing–review and editing; Miles Parkes: data curation; funding acquisition; methodology; project administration; resources; supervision; writing–review and editing; Timothy R Orchard: conceptualisation; resources; supervision; writing–review and editing; Lucy C Hicks: conceptualisation; formal analysis; funding acquisition; investigation; project administration; resources; supervsion; writing–original draft. Horace R T Williams: conceptualisation; formal analysis; funding acquisition; investigation; methodology; project administration; resources; supervision; writing–original draft. SB and LMG had full access to and verified the data.

## Data sharing statement

Patient-level data underlying this study are available to researchers subject to the access processes of the UK IBD BioResource, detailed further at: https://www.ibdbioresource.nihr.ac.uk/index.php/resources. Processed data will be available on reasonable request via contacting the corresponding author.

## Declaration of interests

Dr Balarajah reports grant support from Bowel Research UK, and support for attending meetings and/or travel from Ferring Pharmaceuticals and Dr Falk Pharma. Dr Alexander reports payment or honoraria for lectures, presentations, speakers bureaus, manuscript writing or educational events from Takeda, Abbvie, Pfizer and Johnson & Johnson, and support for attending meetings and/or travel from Takeda, Celltrion, Tillotts Pharma and Lilly. Dr Mullish reports grant support from the Medical Research Council (MRC). Dr Perry reports grant support from Guts UK, and support for attending meetings and/or travel from Ferring Pharmaceuticals. Professor Parkes reports grant support from the Wellcome Trust and the Helmsley Charitable Trust. Professor Williams reports grant support from the Imperial NIHR Biomedical Research Centre, Guts UK and Bowel Research UK, consulting fees from Pfizer, payment or honoraria for lectures, presentations, speakers bureaus, manuscript writing or educational events from Takeda and Tillots Pharma, and support for attending meetings and/or travel from Takeda. The following authors have nothing to declare: Dr Martinez-Gili; Dr Li; Professor Orchard; Dr Hicks.
