# Supplementary material for: A large-scale comparison of clinical outcomes to IBD therapies in White and South Asian ethnicities
Source: eClinicalMedicine. 2025 Nov 18;90:103644. doi: 10.1016/j.eclinm.2025.103644 (PMC12766419; doi:10.1016/j.eclinm.2025.103644)
Supplement: Supplementary Material [file mmc1.docx]

**A large-scale comparison of clinical outcomes to IBD therapies in White and South Asian ethnicities: Supplementary material**

***Balarajah S*^1,2^, Martinez-Gili L*^1^, Alexander JL^1,2,3^, Mullish BH^1,2^, Perry RW^1,2^, Li JV^1^, Marchesi JR^1^, Parkes M^4,5^, Orchard TR^1,2^, Hicks LC^1,2^, Williams HRT^1,2^, UK IBD BioResource Investigators***

**Joint first authors*

^1^ Department of Metabolism, Digestion and Reproduction, Imperial College London, UK

^2^ Department of Gastroenterology and Hepatology, Imperial College Healthcare NHS Trust, UK

^3^ IBD Unit, St Mark’s National Bowel Hospital, London, UK

^4^ Department of Medicine, University of Cambridge, UK

^5^ Department of Gastroenterology, Cambridge University Hospitals NHS Trust, UK

**Supplementary text**

**Methods: Assessment of treatment response**

***Diagnostic era***

Diagnostic era accounted for when drugs were approved for use in the UK and was categorised into three groups: pre-thiopurines (before 1980), thiopurines (1980-1998)^1^, and biologics (1999 and after for CD^2^, 2008 and after for UC^3^).

***Treatment outcomes***

As part of the data collection process for the IBD-BR, the effectiveness of each drug for every participant was assessed via the question, "Was the treatment effective?" with eight possible response categories:

1. Yes
2. No
3. Partial (response but not remission)
4. Worked for <12 months then lost response
5. Worked for >12 months then lost response
6. Not known
7. Only at high dose/increased frequency
8. Unable to assess (e.g. unable to tolerate)

**Supplementary tables**

| Supplementary Table 1  Demographic and phenotypic characteristics of White (WH) and South Asian (SA) UC patients treated with 5-ASAs | | | |
| --- | --- | --- | --- |
|  | **WH**  **N = 8539** | **SA**  **N = 420** | ***p*** |
| Age (years) at diagnosis,  Median (IQR) | 36 (26-50) | 30 (23-38) | *<0.001**** |
| Age (years) at commencing 5-ASA,  Median (IQR) | 39 (28-53) | 32 (24-42) | *<0.001**** |
| Sex, N (%) |  |  | *<0.001**** |
| Male | 4175 (48.9) | 245 (58.3) | *_* |
| Female | 4364 (51.1) | 175 (41.7) | *_* |
| Smoking status at diagnosis, N (%) |  |  | *<0.001**** |
| Never smoked | 3702 (45.2) | 304 (76.6) | *_* |
| Ex-smoker | 3952 (48.2) | 73 (18.4) | *_* |
| Current smoker | 543 (6.6) | 20 (5.0) | *_* |
| Co-morbidity, N (%) | 2823 (33.1) | 64 (15.2) | *<0.001**** |
| History of EIM, N (%) | 888 (10.9) | 32 (8.0) | *0.08* |
| Disease extent, N (%) |  |  | *<0.001**** |
| Proctitis | 1538 (19.9) | 47 (13.2) | *_* |
| Left-sided | 3807 (49.3) | 164 (46.1) | *_* |
| Extensive | 2380 (30.8) | 145 (40.7) | *_* |
| Treatment era, N (%) |  |  | *0.07* |
| Thiopurines era | 2988 (36.1) | 132 (31.6) | *_* |
| Biologics era | 5290 (63.9) | 286 (68.4) | *_* |
| Time (years) from diagnosis to commencing 5-ASA, N (%) |  |  | *0.28* |
| < 1 year | 6015 (70.4) | 306 (72.9) | *_* |
| ≥ 1 year and < 2 years | 705 (8.3) | 38 (9.0) | *_* |
| ≥ 2 years | 1819 (21.3) | 76 (18.1) | *_* |

| Supplementary Table 2  Demographic and phenotypic characteristics of White (WH) and South Asian (SA) CD patients treated with thiopurines | | | |
| --- | --- | --- | --- |
|  | **WH**  **N = 7611** | **SA**  **N = 224** | ***p*** |
| Age (years) at diagnosis,  Median (IQR) | 25 (18-37) | 25 (17-36) | *0.19* |
| Age (years) at commencing thiopurine, Median (IQR) | 32 (22- 46) | 29.0 (19-40) | *<0.001**** |
| Sex, N (%) |  |  | *<0.001**** |
| Male | 3458 (45.4) | 139 (62.1) | *_* |
| Female | 4153 (54.6) | 85 (37.9) | *_* |
| Smoking status at diagnosis, N (%) |  |  | *<0.001**** |
| Never smoked | 3365 (47.0) | 159 (74.6) | *_* |
| Ex-smoker | 1217 (17.0) | 25 (11.7) | *_* |
| Current smoker | 2573 (36.0) | 29 (13.6) | *_* |
| Co-morbidity, N (%) | 2123 (27.9) | 34 (15.2) | *<0.001**** |
| History of EIM, N (%) | 1485 (20.6) | 30 (14.4) | *0.04** |
| Disease location, N (%) |  |  | *0.59* |
| Ileal | 2776 (37.5) | 73 (34.8) | *_* |
| Colonic | 1881 (25.4) | 52 (24.8) | *_* |
| Ileocolonic | 2661 (36.0) | 81 (38.6) | *_* |
| Isolated upper GI | 83 (1.1) | 4 (1.9) | *_* |
| Behaviour, N (%) |  |  | *0.006*** |
| Inflammatory | 4354 (60.6) | 146 (71.6) | *_* |
| Stricturing | 884 (12.3) | 17 (8.3) | *_* |
| Penetrating | 1951 (27.1) | 41 (20.1) | *_* |
| Perianal involvement, N (%) | 2356 (32.6) | 80 (39.0) | *0.06* |
| Treatment era, N (%) |  |  | *<0.001**** |
| Pre-thiopurines era | 275 (3.6) | 2 (0.9) | *_* |
| Thiopurines era | 1666 (21.9) | 19 (8.5) | *_* |
| Biologics era | 5670 (74.5) | 203 (90.6) | *_* |
| Time (years) from diagnosis to commencing thiopurine, N (%) |  |  | *0.22* |
| < 1 year | 2595 (34.1) | 88 (39.3) | *_* |
| ≥ 1 year and < 2 years | 1307 (17.2) | 39 (17.4) | *_* |
| ≥ 2 years | 3709 (48.7) | 97 (43.3) | *_* |

| Supplementary Table 3  Demographic and phenotypic characteristics of White (WH) and South Asian (SA) UC patients treated with thiopurines | | | |
| --- | --- | --- | --- |
|  | **WH**  **N = 5147** | **SA**  **N = 300** | ***p*** |
| Age (years) at diagnosis,  Median (IQR) | 39 (27-52) | 32 (23-40) | *<0.001** |
| Age (years) at commencing thiopurine, Median (IQR) | 39 (27-52) | 32 (23-40) | *<0.001**** |
| Sex, N (%) |  |  | *0.01* |
| Male | 2732 (53.1) | 182 (60.7) | *_* |
| Female | 2415 (46.9) | 118 (39.3) | *_* |
| Smoking status at diagnosis, N (%) |  |  | *<0.001**** |
| Never smoked | 2404 (49.2) | 222 (79.9) | *_* |
| Ex-smoker | 2210 (45.2) | 41 (14.7) | *_* |
| Current smoker | 276 (5.6) | 15 (5.4) | *_* |
| Co-morbidity, N (%) | 1573 (30.6) | 50 (16.7) | *<0.001**** |
| History of EIM, N (%) | 677 (13.8) | 34 (11.8) | *0.39* |
| Disease extent, N (%) |  |  | *0.15* |
| Proctitis | 436 (9.4) | 17 (6.7) | *_* |
| Left-sided | 2268 (49.0) | 119 (46.7) | *_* |
| Extensive | 1924 (41.6) | 119 (46.7) | *_* |
| Treatment era, N (%) |  |  | *0.03** |
| Pre-thiopurines | 109 (2.1) | 3 (1.0) | *_* |
| Thiopurines era | 2145 (41.7) | 106 (35.3) | *_* |
| Biologics era | 2893 (56.2) | 191 (63.7) | *_* |
| Time (years) from diagnosis to commencing thiopurine, N (%) |  |  | *0.03** |
| < 1 year | 1244 (24.2) | 86 (28.7) | *_* |
| ≥ 1 year and < 2 years | 987 (19.2) | 67 (22.3) | *_* |
| ≥ 2 years | 2916 (56.7) | 147 (49.0) | *_* |

| Supplementary Table 4  Demographic and phenotypic characteristics of White (WH) and South Asian (SA) CD patients treated with anti-TNFs | | | |
| --- | --- | --- | --- |
|  | **WH**  **N = 7736** | **SA**  **N = 239** | ***p*** |
| Age (years) at diagnosis,  Median (IQR) | 24 (18-34) | 22 (15-34) | *0.004* |
| Age (years) at commencing anti-TNF, Median (IQR) | 33 (24-47) | 28 (20-39) | *<0.001**** |
| Sex, N (%) |  |  | *<0.001**** |
| Male | 3664 (47.4) | 164 (68.6) | *_* |
| Female | 4072 (52.6) | 75 (31.4) | *_* |
| Smoking status at diagnosis, N (%) |  |  | *<0.001**** |
| Never smoked | 3582 (49.2) | 160 (71.4) | *_* |
| Ex-smoker | 2440 (33.5) | 36 (16.1) | *_* |
| Current smoker | 1258 (17.3) | 28 (12.5) | *_* |
| Co-morbidity, N (%) | 1967 (25.4) | 25 (10.5) | *<0.001**** |
| History of EIM, N (%) | 5528 (76.3) | 173 (80.5) | *0.18* |
| Disease location, N (%) |  |  | *0.04** |
| Ileal | 2489 (33.0) | 54 (24.1) | *_* |
| Colonic | 2042 (27.1) | 67 (29.9) | *_* |
| Ileocolonic | 2910 (38.6) | 101 (45.1) | *_* |
| Isolated upper GI | 99 (1.3) | 2 (0.9) | *_* |
| Behaviour, N (%) |  |  | *0.003*** |
| Inflammatory | 4352 (59.8) | 153 (71.5) | *_* |
| Stricturing | 1925 (26.5) | 40 (18.7) | *_* |
| Penetrating | 995 (13.7) | 21 (9.8) | *_* |
| Perianal involvement, N (%) | 2913 (39.9) | 105 (47.7) | *0.02** |
| Time (years) from diagnosis to commencing anti-TNF, N (%) |  |  | *0.90* |
| < 1 year | 1254 (16.2) | 40 (16.7) | *_* |
| ≥ 1 year and < 2 years | 1131 (14.6) | 37 (15.5) | *_* |
| ≥ 2 years | 5351 (69.2) | 162 (67.8) | *_* |
| Concomitant immunomodulator, N (%) | 4762 (78.6) | 150 (81.5) | *0.39* |

| Supplementary Table 5  Demographic and phenotypic characteristics of White (WH) and South Asian (SA) UC patients treated with anti-TNFs | | | |
| --- | --- | --- | --- |
|  | **WH**  **N = 3218** | **SA**  **N = 204** | ***WH vs SA***  ***p*** |
| Age (years) at diagnosis,  Median (IQR) | 31 (22-44) | 28 (21-35) | *<0.001**** |
| Age (years) at commencing anti-TNF, Median (IQR) | 38 (28-52) | 33 (25-41) | *<0.001**** |
| Sex, N (%) |  |  | *0.48* |
| Male | 1693 (52.6) | 113 (55.4) | *_* |
| Female | 1525 (47.4) | 91 (44.6) | *_* |
| Smoking status at diagnosis, N (%) |  |  | *<0.001**** |
| Never smoked | 1467 (48.5) | 154 (79.4) | *_* |
| Ex-smoker | 177 (5.8) | 11 (5.7) | *_* |
| Current smoker | 1383 (45.7) | 29 (14.9) | *_* |
| Co-morbidity, N (%) | 793 (24.6) | 28 (13.7) | *0.001*** |
| History of EIM, N (%) | 2559 (85.4) | 168 (87.0) | *0.61* |
| Disease extent, N (%) |  |  | *0.71* |
| Proctitis | 222 (7.8) | 12 (7.0) | *_* |
| Left-sided | 1489 (52.1) | 85 (49.7) | *_* |
| Extensive | 1147 (40.1) | 74 (43.3) | *_* |
| Time (years) from diagnosis to commencing anti-TNF, N (%) |  |  | *0.95* |
| < 1 year | 544 (16.9) | 33 (16.2) | *_* |
| ≥ 1 year and < 2 years | 485 (15.1) | 32 (15.7) | *_* |
| ≥ 2 years | 2189 (68.0) | 139 (68.1) | *_* |
| Concomitant immunomodulator, N (%) | 1873 (80.8) | 110 (71.0) | *0.004*** |
| ** p <0.05, ** p <0.001, *** p <0.001*  *WH, White; SA, South Asian* |  |  |  |

| Supplementary Table 6 Cox regression analysis of treatment effectiveness in CD | | | | |
| --- | --- | --- | --- | --- |
|  | **Thiopurines**  **N = 7835** | | **Anti-TNFs (IFX, ADA)**  **N = 7975** | |
|  | **HR (95% CI)** | ***p*** | **HR (95% CI)** | ***p*** |
| Age at commencing therapy | 1.01 (1.01-1.02) | *<0.001**** | 1.01 (1.00-1.01) | *0.20* |
| Time from diagnosis to treatment initiation | 1.04 (1.03-1.05) | *<0.001**** | 1.00 (0.98-1.01) | *0.64* |
| Sex |  |  |  |  |
| Male (reference group) | _ | *_* | _ | *_* |
| Female | 1.09 (0.95-1.25) | *0.22* | 1.18 (0.99-1.41) | *0.07* |
| Smoking status at diagnosis |  |  |  |  |
| Never smoker (reference group) | _ | *_* | _ | *_* |
| Ex-smoker | 1.15 (0.98-1.34) | *0.08* | 1.11 (0.91-1.35) | *0.31* |
| Current smoker | 1.10 (0.91-1.33) | *0.31* | 1.08 (0.86-1.36) | *0.51* |
| Co-morbidity | 0.98 (0.84-1.14) | *0.81* | 1.07 (0.88-1.30) | *0.50* |
| History of EIM | 1.07 (0.92-1.26) | *0.38* | 0.74 (0.61-0.89) | *0.001*** |
| Disease extent |  |  |  |  |
| L1 (reference group) | _ | *_* | _ | *_* |
| L2 | 1.19 (0.95-1.49) | *0.14* | 0.97 (0.72-1.29) | *0.81* |
| L3 | 0.99 (0.86-1.15) | *0.94* | 1.09 (0.89-1.33) | *0.41* |
| L4 | 0.85 (0.40-1.82) | *0.68* | 0.23 (0.07-0.76) | *0.02** |
| Perianal involvement | 1.03 (0.90-1.19) | *0.66* | 0.89 (0.74-1.07) | *0.22* |
| Disease behaviour |  |  |  |  |
| Inflammatory (reference group) | _ | *_* | _ | *_* |
| Stricturing | 1.02 (0.87-1.19) | *0.85* | 1.06 (0.87-1.29) | *0.58* |
| Penetrating | 1.00 (0.83-1.20) | *0.96* | 1.06 (0.85-1.34) | *0.59* |
| Previous surgery | 0.86 (0.73-1.02) | *0.09* | 0.95 (0.78-1.17) | *0.66* |
| Concomitant immunomodulator | _ | *_* | 0.61 (0.50-0.74) | *<0.001**** |
| Adverse event with therapy | 2.14 (1.86-2.46) | *<0.001**** | 1.45 (1.20-1.75) | *<0.001**** |
| ** p <0.05, ** p<0.001, *** p<0.001*  *WH, White; SA, South Asian; EIM, extraintestinal manifestation*  *Adjusted for age at commencing therapy, ethnicity, disease duration, diagnostic era (thiopurines), sex, smoking status, co-morbidities, extraintestinal manifestations, disease extent, disease behaviour, perianal involvement, previous surgery, concurrent corticosteroid use, concomitant immunomodulator use (anti-TNFs) and adverse events* | | | | |

| Supplementary Table 7 Cox regression analysis of treatment effectiveness in UC | | | | | | |
| --- | --- | --- | --- | --- | --- | --- |
|  | **5-ASAs**  **N = 8959** | | **Thiopurines**  **N = 5447** | | **Anti-TNFs (IFX, ADA, GOL)**  **N = 3422** | |
|  | **HR (95% CI)** | ***p*** | **HR (95% CI)** | ***p*** | **HR (95% CI)** | ***p*** |
| Age at commencing therapy | 1.00 (1.00-1.01) | *0.03** | 1.01 (1.00-1.01) | *0.009*** | 1.01 (1.00-1.02) | *0.04** |
| Time from diagnosis to treatment initiation | 1.07 (1.05-1.09) | *<0.001**** | 1.06 (1.05-1.08) | *<0.001**** | 0.98 (0.96-0.99) | *0.008** |
| Sex |  |  |  |  |  |  |
| Male (reference group) | _ | *_* | _ | *_* | _ | *_* |
| Female | 0.95 (0.84-1.08) | *0.44* | 0.97 (0.86-1.10) | *0.64* | 1.04 (0.85-1.27) | *0.73* |
| Smoking status at diagnosis |  |  |  |  |  |  |
| Never smoker (reference group) | _ | *_* | _ | *_* | _ | *_* |
| Ex-smoker | 0.92 (0.81-1.05) | *0.23* | 0.93 (0.81-1.06) | *0.25* | 0.98 (0.79-1.20) | *0.82* |
| Current smoker | 1.27 (0.95-1.70) | *0.11* | 1.15 (0.86-1.55) | *0.35* | 0.71 (0.46-1.11) | *0.14* |
| Co-morbidity | 0.95 (0.83-1.08) | *0.43* | 0.99 (0.86-1.14) | *0.91* | 0.93 (0.74-1.18) | *0.56* |
| History of EIM | 0.89 (0.76-1.05) | *0.18* | 1.16 (0.98-1.36) | *0.08* | 0.97 (0.74-1.26) | *0.81* |
| Disease extent |  |  |  |  |  |  |
| E1 (reference group) | _ | *_* | _ | *_* | _ | *_* |
| E2 | 0.78 (0.61-0.98) | *0.03** | 0.96 (0.76-1.22) | *0.76* | 1.27 (0.84-1.91) | *0.26* |
| E3 | 0.81 (0.63-1.03) | *0.08* | 0.90 (0.71-1.15) | *0.40* | 1.11 (0.74-1.69) | *0.61* |
| Concomitant immunomodulator | _ | *_* | _ | *_* | 0.68 (0.54-0.86) | *0.001*** |
| Adverse event with therapy | 2.04 (1.69-2.46) | *<0.001**** | 2.72 (2.39-3.09) | *<0.001**** | 1.11 (0.89-1.38) | *0.37* |
| ** p <0.05, ** p<0.001, *** p<0.001*  *WH, White; SA, South Asian; EIM, extraintestinal manifestation*  *Adjusted for age at commencing therapy, ethnicity, disease duration, diagnostic era (5-ASAs and thiopurines), sex, smoking status, co-morbidities, extraintestinal manifestations, disease extent, concurrent corticosteroid use, concomitant immunomodulator use (anti-TNFs) and adverse events* | | | | | | |

| Supplementary Table 8  Demographic and phenotypic characteristics of White and South Asian IBD patients assessed for adverse events on first exposure to 5-ASAs, thiopurines and anti-TNFs | | | | | | | | | |
| --- | --- | --- | --- | --- | --- | --- | --- | --- | --- |
|  | **5-ASAs (UC only)** | | | **Thiopurines** | | | **Anti-TNFs** | | |
|  | **WH** | **SA** | ***p*** | **WH** | **SA** | ***p*** | **WH** | **SA** | ***p*** |
| Total, N (%) | 11688 (95.2) | 583 (4.8) | *_* | 13905 (96.1) | 560 (3.9) | *_* | 9324 (96.2) | 372 (3.8) | *_* |
| Age (years) at commencing therapy, Median (IQR) | 39 (28-53) | 31 (23-42) | *<0.001**** | 34 (24-47) | 31 (21-40) | *<0.001**** | 26 (19-39) | 27 (18-35) | *0.03** |
| Sex, N (%) |  |  | *<0.001**** |  |  | *<0.001**** |  |  | *<0.001**** |
| Male | 5823 (49.8) | 338 (58.0) | *_* | 6943 (49.9) | 349 (62.3) | *_* | 4665 (50.0) | 231 (62.1) | *_* |
| Female | 5865 (50.2) | 245 (42.0) | *_* | 6962 (50.1) | 211 (37.7) | *_* | 4659 (50.0) | 141 (37.9) | *_* |
| IBD subtype, N (%) |  |  | *_* |  |  | *<0.001**** |  |  | *<0.001**** |
| CD | _ | _ | *_* | 8707 (62.6) | 255 (45.5) | *_* | 6384 (68.5) | 180 (48.4) | *_* |
| UC | _ | _ | *_* | 5198 (37.4) | 305 (54.5) | *_* | 2940 (31.5) | 192 (51.6) | *_* |
| Smoking status at diagnosis, N (%) |  |  | *<0.001**** |  |  | *<0.001**** |  |  | *<0.001**** |
| Never smoked | 5170 (46.6) | 425 (77.7) | *_* | 6417 (48.8) | 404 (77.2) | *_* | 4325 (49.3) | 258 (73.5) | *_* |
| Ex-smoker | 5229 (47.1) | 95 (17.4) | *_* | 5061 (38.5) | 80 (15.3) | *_* | 3244 (37.0) | 62 (17.7) | *_* |
| Current smoker | 698 (6.3) | 27 (4.9) | *_* | 1672 (12.7) | 39 (7.5) | *_* | 1202 (13.7) | 31 (8.8) | *_* |
| Co-morbidity, N (%) | 3773 (32.3) | 90 (15.4) | *<0.001**** | 3860 (27.8) | 87 (15.5) | *<0.001**** | 2303 (24.7) | 46 (12.4) | *<0.001**** |
| History of EIM, N (%) | 1300 (11.7) | 58 (10.3) | *0.37* | 2320 (17.6) | 69 (13.2) | *0.01** | 1660 (19.1) | 57 (16.5) | *0.26* |
| ** p <0.05, ** p <0.001, *** p <0.001*  *WH, White; SA, South Asian; EIM, extraintestinal manifestation* | | | | | | | | | |

| Supplementary Table 9  Adverse events to 5-ASAs | | |
| --- | --- | --- |
|  | **WH**  **N=11688** | **SA**  **N=583** |
| Any adverse event, N (%) | 1005 (8.6) | 51 (8.7) |
| Breakdown of adverse events, N (%) |  |  |
| Deranged liver function tests | 31 (0.3) | 0 (0.0) |
| Interstitial nephritis | 11 (0.1) | 0 (0.0) |
| Joint pain | 46 (0.4) | 4 (0.7) |
| Leucopenia | 10 (0.1) | 0 (0.0) |
| Pancreatitis | 16 (0.1) | 1 (0.2) |
| Pancytopenia | 3 (0.0) | 0 (0.0) |
| Rash | 73 (0.6) | 3 (0.5) |
| Sepsis | 1 (0.0) | 1 (0.2) |
| Other | 612 (5.2) | 33 (5.7) |
| *WH, White; SA, South Asian* | | |

| Supplementary Table 10  Adverse events to thiopurines |  |  |
| --- | --- | --- |
|  | **WH**  **N=13905** | **SA**  **N=372** |
| Any adverse event, N (%) | 1009 (10.8) | 46 (12.4) |
| Breakdown of adverse events, N (%) |  |  |
| Anaphylaxis | 28 (0.2) | 0 (0.0) |
| Deranged liver function tests | 540 (3.9) | 13 (2.3) |
| Fever | 67 (0.5) | 3 (0.5) |
| Flu-like symptoms | 202 (1.5) | 6 (1.1) |
| Joint pain | 200 (1.4) | 12 (2.1) |
| Leucopenia | 256 (1.8) | 21 (3.8) |
| Pancreatitis | 319 (2.3) | 20 (3.6) |
| Panyctopenia | 28 (0.2) | 2 (0.4) |
| Rash | 215 (1.5) | 4 (0.7) |
| Sepsis | 14 (0.1) | 1 (0.2) |
| Other | 2373 (17.1) | 97 (17.3) |
| *WH, White; SA, South Asian* | | |

| Supplementary Table 11  Adverse events to anti-TNFs | | |
| --- | --- | --- |
|  | **WH**  **N=9324** | **SA**  **N=372** |
| Any adverse event, N (%) | 1009 (10.8) | 46 (12.4) |
| Breakdown of adverse events, N (%) |  |  |
| Anaphylaxis | 73 (0.8) | 5 (1.3) |
| Demyelination/other neurological symptoms | 29 (0.3) | 1 (0.3) |
| Deranged liver function tests | 22 (0.2) | 1 (0.3) |
| Hypertension | 1 (0.0) | 0 (0.0) |
| Renal impairment | 37 (0.4) | 6 (1.6) |
| Joint pain | 103 (1.1) | 4 (1.1) |
| Panyctopenia | 4 (0.0) | 0 (0.0) |
| Psoriasis | 46 (0.5) | 0 (0.0) |
| Rash | 97 (1.0) | 5 (1.3) |
| Sepsis | 5 (0.1) | 0 (0.0) |
| Other | 575 (6.2) | 26 (7.0) |
| *WH, White; SA, South Asian* | | |

**References**

1. Manu P, Rogozea LM, Dumitraşcu DL. Pharmacological management of inflammatory bowel disease: A century of expert opinions in cecil textbook of medicine. *Am J Ther* 2022;**29**:e500-e6.

2. European medicines agency: Remicade. <https://www.ema.europa.eu/en/medicines/human/EPAR/remicade#ema-inpage-item-overview> Accessed 7th February.

3. Nice infliximab for acute exacerbations of ulcerative colitis. <https://www.nice.org.uk/guidance/ta163/chapter/2-The-technology> Accessed 7th February, 2008.
